# Supplementary material for: Toward the Definition of a Repertoire of Technical Professional Specialist Competencies for Operating Room Nurses: An Ethnographic Study
Source: Healthcare (Basel). 2024 Sep 5;12(17):1774. doi: 10.3390/healthcare12171774 (PMC11394865; doi:10.3390/healthcare12171774)
Supplement: Supplementary file 1 [file healthcare-12-01774-s001.zip › healthcare-3142258-supplementary.pdf]

Supplementary material

Table S1: Open-questions used in the team meeting discussions

✔ Which competencies should we differentiate between for perioperative and perianesthesiological nursing, and which should we combine?<br>✔ Are the activities related to documenting nursing care planned and delivered in the perianesthesia setting by the Scrub Nurse also performed in the perianesthesia setting by the Anesthesia and Recovery Room Nurses?<br>✔ Should the competency of preparing and restoring the perioperative and perianesthesiological environment be further subdivided into preparation and restoration?<br>✔ Can activities included in perioperative nursing also apply to perianesthesiological nursing? If so, how do they differ for the roles of Scrub Nurse, Anesthesia Nurse, and Recovery Room Nurse?<br>✔ Can you elaborate on this task by describing it in more detail?<br>✔ Which verb is most suitable for describing this specific task?<br>✔ According to the activity, which competency should it be associated with? If it falls under two competencies, which role does it belong to?<br>✔ Are the competencies overlapping, or is one preferable to the other?<br>✔ If the Post-it notes were very brief (not clear or complete), could you please clarify what was meant by the content?<br>✔ What verbs should be used to describe the tasks in relation to the activities?

Table S2: Open-questions used in the "Interview to the double"

Attention<br>In the questions, it is often necessary to remind the interviewee that the interviewer will be a "look-alike" (or "doppelgänger"). Therefore, the term has been intentionally repeated multiple times in the questions.
Initial Guide<br>If tomorrow I were to replace you in the operating room, taking your place as a look-alike (your double), what activities would I need to perform? Describe to me in detail the activities and tasks I should undertake, including the specific behaviors I need to exhibit, so that your colleagues do not realize that I am substituting you.
Questions
✔ If I were in your place, what should I do to prepare the perioperative environment?<br>✔ What information should I verify in the operative note? Please indicate which essential details I must collect if I were in your place.<br>✔ If I were in your place, could I be assigned to that operating room in the role of anesthesia nurse, even if I was previously informed that I would be filling the role of scrub nurse?<br>✔ Once I have obtained all the necessary information, what should I do if I were in your place?<br>✔ Are there any other important pieces of information that I should be aware of?<br>✔ Can you specify the medical devices that I need to check for presence, integrity, and functionality?<br>✔ After verifying that the medical devices are present and functioning, how should I proceed?
✔ If tomorrow I were in your place, what should I do to prepare the perianesthesia environment?<br>✔ After verifying that the medical devices just mentioned are present and functioning, what should I do if I were in your place?<br>✔ If tomorrow I were in your place, what should I do to prepare the patient for the surgical procedure?<br>✔ Considering the presence of the anesthesia nurse who will prepare the patient for anesthesia,

what precautions do you suggest I take to avoid interfering with my colleague's work?

- ✔ In your place tomorrow, to prepare the patient for anesthesia, what do you suggest I do? Are the checks you performed as a scrub nurse largely similar to those done by the anesthesia nurse?
- ✔ And how does the preparation I will need to handle as an anesthesia nurse differ?

Table S3 Themes and subthemes identified in the interview to the double.

Theme	Subthemes	Codes
Nursing care in routine and emergency setting	Perianaesthesiological nursing care	Access to the operating rooms
		Application of the safety checklist
		Execution of procedures
		Health education
		Management of biological material
		Positioning of the operating field
		Positioning of the surgical team
		Verification of the safety checks
	Perioperative nursing care	Access to the operating rooms
		Application of the safety checklist
		Execution of the procedures
		Health education
		Management of biological material
		Positioning of the operating field
		Positioning of the surgical team
		Verification of the safety checks
Operating room environment and resources	Perianaesthesiological environment	Access to structural resources
		Access to the operating room
		Antiseptic hand washing
		Checking the operating list
		Disposal due to expiration
		Ensuring asepsis during procedures
		Maintenance request

Theme	Subthemes	Codes
		Restocking of materials
		Restoring material and technological resources
		Setup of medical devices and electro-medical equipment
		Supervision of access to the rooms
		Use of material and technological resources
		Verification of the availability of resources for use
		Verification of the functionality and integrity of resources
	Perioperative environment	Access to structural resources
		Access to the rooms
		Checking the operating list
		Dispatch of materials
		Disposal in case of expiration
		Dressing
		Ensuring asepsis
		Maintenance request
		Restoring material and technological resources
		Setup of medical devices and electro-medical equipment
		Supervision of access
		Surgical hand washing
		Verification of availability and reusability of resources
		Verification of functionality and integrity of resources
Patient care in the perioperative and perianaesthesiological environment	Admitting the patient	Admission registration
		Health condition verification
		Information gathering

Theme	Subthemes	Codes
		Patient reception
		Review of operative notes
		Verification of useful information for surgical procedures
	Position the assisted person for surgery	Collaboration with the team during procedures
		Promotion of comfort and privacy
		Verification of the functionality of the surgical positioning
		Verification of the functioning of the patient monitoring system
	Preparation for anesthesiological procedures	Monitoring of the patient
		Peri-anesthesiological health education
		Preparation of the patient
		Preparation of the patient for procedures
		Verification of procedures in the checklist
		Verification of the surgical site and laterality
	Preparation for surgical procedures	Antisepsis of the surgical site
		Collaboration with the team during procedures
		Positioning on the operating table
		Preparation of the patient for surgical procedures
		Verification of procedures with the checklist
		Verification of the surgical site and laterality
	Take care the assisted person	Collaboration with the team during procedures
		Patient information
		Safety checklist control
		Satisfaction of the patient's needs
		Team collaboration in risk assessment

Theme	Subthemes	Codes
	Transferring the assisted person	Ensuring the functionality and integrity of the operating table and devices
		Team collaboration in selecting the operating table and necessary devices
		Team collaboration in transferring the patient throughout the process
Promotion of Nursing Care Continuity and Clinical Governance	Documenting planned and delivered nursing care	Collaboration in completing the anesthesiology record
		Collaboration in completing the operating room record
		Documentation of the anesthesia nursing activities
		Documentation of the planning of nursing activities
		Use of models, tools, and standardized language for documenting in the nursing record
		Verification and archiving of documentation
		Verification of the completeness of anesthesiological procedure documentation
		Verification of the completeness of the hospital documentation
	Perianaesthesiological Safety and Risk Management	Collaborate in the completion of peri-anesthesiological documentation
		Collaborate in the identification of peri-anesthesiological risks
		Ensure patient safety
		Verify the safety of the anesthesiological environment
		Verify the safety of the peri-anesthesiological context
	Perianesthesiological care continuity	Complete the peri-anesthesiological care continuity form
		Gather information for anesthesiological management
		Plan peri-anesthesiological care
		Share necessary information with the team
		Verify the care continuity record
	Perioperative care continuity	Collect data on the nursing management of instruments
		Complete the perioperative care continuity form

Theme	Subthemes	Codes
		Plan peri-operative care
		Share necessary information for the surgical procedures
		Verify the care continuity record
	Perioperative Safety and Risk Management	Assist in the completion of perioperative documentation
		Collaborate with the team in identifying risks
		Ensure perioperative safety
		Verify the safety of the perioperative context
		Verify the safety of the perioperative environment
	Professional development, competencies enhancement, and best practices	Act based on your level of experience
		Adhere to guidelines
		Commit to continuing education
		Recognize the value of patient safety
		Recognize the value of research
		Recognize the value of training

Table S4. Repertoire of technical-professional competencies of perioperative and perianesthesiological nursing

AREA of ACTIVITY 1	PREPARATION AND RESTORATION OF THE PERIOPERATIVE, PERIANAESTHESIOLOGICAL ENVIRONMENT AND MATERIAL AND TECHNOLOGICAL RESOURCES
**Competency 1** Preparing the perioperative environment	**Learning outcome 1**<br>Accessing, orienting and positioning oneself within the structural, environmental and organisational peri-operative context, using the dedicated spaces and paths, as well as the material and technological resources necessary for instrumentation activities, setting up, preparing and restoring furniture, medical devices and electro-medical equipment, complying with the behavioural and good practice recommendations, taking care of the relationships established with the human resources involved
	**Activities/tasks**
	1. Accessing the context of the operating blocks and the context of the Preparation Room and the referring O.S., using clean and dirty paths and afferent spaces appropriately, interacting effectively with the operating theatre team;<br>2. Checking the operating list to acquire information regarding the person assisted and the surgical procedure, taking into account any changes in the note and/or all possible problems/complications that may occur during the performance of instrument-related activities (e.g. latex allergies or massive blood loss, etc.), transmitting the information that

	emerges from time to time to the entire operating theatre team;<br>3. Verifying in the relevant O.S., also according to checklists where present, that all furniture, medical devices and electro-medical equipment necessary for instrumentation activities are present, undamaged and in working order (completely listed in the full version of the document);<br>4. Proceeding with the maintenance request and dispatch of material and technological resources to the relevant offices, proceeding with disposal in the event of expiry;<br>5. Performing surgical hand washing, then the dress-up, and using the material and technological resources necessary for instrumentation activities, setting up and arranging furniture, medical devices and electro-medical equipment, continuously complying with the principles of disinfection, sanitisation and sterilisation and the time schedules;<br>6. Supervising access to the operating theatre and the movements performed by all members of the surgical and anaesthesiologic team, ensuring that asepsis is maintained during all procedures to prepare the peri-operative environment;
**Competency 2**<br>Preparing the perianaesthesiological environment	**Learning outcome 2**<br>Accessing, orienting and positioning oneself within the structural, environmental and organisational peri-operative context, using the dedicated spaces and pathways, as well as the material and technological resources necessary for the activity of anaesthesiologic conduct, setting up, arranging and restoring furniture, medical devices and electro-medical equipment, complying with the behavioural and good practice recommendations, taking care of the relationships established with the human resources involved
	**Activities/tasks**
	1. Access the context of the operating blocks and the context of the Preparation Room, the referral O.S. and the Recovery Room, appropriately using the clean and dirty paths and afferent spaces, interacting effectively with the operating theatre team;<br>2. Check the operating list to acquire information regarding the person assisted, the anaesthesiologic conduct and the surgical procedure, taking into account any changes in the note and/or all possible problems/complications that may occur during the performance of activities related to the anaesthesiologic conduct (e.g. latex allergies or massive blood loss, malignant hyperthermia or ab ingestis in the case of full stomach intubation, etc.), passing on the information that emerges from time to time to the entire operating theatre team;<br>3. Check, also according to check lists where present, both in the Preparation Room and in the O.R. of reference and in the Recovery Room, that the narcotic and non-narcotic drugs necessary to carry out the nursing and medical procedures related to the different types of anaesthesia agreed upon are present, that they have not expired, and furthermore that all the furnishings, medical devices and electro-medical equipment necessary for anaesthesiologic conduct are present, intact and functioning (completely reported in the full version of the document);<br>4. Proceed with the maintenance request and dispatch of material and technological resources to the relevant offices, proceeding with disposal in the event of expiry;<br>5. Perform antiseptic hand washing and utilises the material and technological resources required for anaesthesiologic conduct, setting up and arranging furniture, medical devices and electro-medical equipment, continuously observing the principles of disinfection, sanitisation and sterilisation and the scheduled times;<br>6. Supervise the access to the operating theatre and the movements performed by all members of the surgical and anaesthesiologic team, ensuring that asepsis is maintained during all procedures to prepare the peri-anaesthesia environment;
**Competency 3**<br>Restoring the perioperative environment	**Learning outcome 3**<br>Accessing, orienting and positioning oneself within the structural, environmental and organisational peri-operative context, using the dedicated spaces and paths, as well as the material and technological resources necessary for instrumentation activities, setting up, preparing and restoring furniture, medical devices and electro-medical equipment, complying with the behavioural and good practices recommendations, taking care of the relationships established with the human resources involved
	**Activities/tasks**
	1. Access the context of the operating blocks and the context of the Preparation Room and the referring O.S., appropriately using the clean and dirty pathways and afferent spaces, interacting effectively with the operating theatre team;<br>2. Restore the material and technological resources made available for the instrumentation of the surgery that has just been completed, i.e. the furnishings, medical devices and

	electro-medical equipment used (completely reported in the full version of the document);<br>3. Verify in the reference O.S., also according to checklists where present, that all furniture, medical devices and electro-medical equipment used for instrumentation activities are available and reusable for the following surgery;<br>4. Provide in the relevant O.S. for the replenishment of the material and technological resource stocks necessary for the next instrumentation activity;<br>5. Proceed with the supply request or notify those in charge of the need if stocks in the dedicated depots are exhausted;<br>6. Proceed with the maintenance request and material and technological resources dispatch to the relevant offices, provide disposal in case of expiry;<br>7. Operate, cooperate and collaborate in the correct recording of biological material taken and send it to the diagnostic services for the necessary processes;<br>8. Complete the relevant S.O's Closure Check List;
**Competency 4**<br>Restoring the Perianaesthesiological environment	**Learning outcome 4**<br>Accessing, orienting and positioning oneself within the structural, environmental and organisational peri-operative context, using the dedicated spaces and pathways, as well as the material and technological resources necessary for the activity of anaesthesiologic conduct, setting up, arranging and restoring furniture, medical devices and electro-medical equipment, complying with the behavioural and good practice recommendations, taking care of the relationships established with the human resources involved
	**Activities/tasks**
	1. Access the context of the operating blocks and the context of the Preparation Room, the referral O.S. and the Recovery Room, appropriately using the clean and dirty pathways and afferent spaces, interacting effectively with the operating theatre team;<br>2. Restore the material and technological resources made available for the anaesthesiologic conduction activity that has just ended, i.e. the furniture, medical devices and electro-medical equipment used, (full details are given in the full version of the document);<br>3. Verify in the Preparation Room, in the reference O.S. and in the Recovery Room, also according to Check Lists where present, that all the furniture, medical devices and electro-medical equipment used for anaesthesiologic conduct are available and reusable for the following surgery;<br>4. Provide in the Preparation Room, in the referring O.S. and in the Recovery Room for the replenishment of the stocks of material and technological resources necessary for the subsequent anaesthesiologic conduct activity;<br>5. Proceed with the supply request or inform those in charge of the need, if stocks in the dedicated depots are exhausted;<br>6. Proceed with the maintenance request and dispatch of material and technological resources to the relevant offices, proceed with disposal in the event of expiry;<br>7. Operate, cooperate and collaborate in the correct recording of biological material taken and send them to diagnostic services for the necessary processes (according to guidelines, protocols, procedures and operating instructions);<br>8. Complete the S.O.'s Closure Check List;
**AREA of ACTIVITY 2**	**RECEPTION, TRANSFER, AND SURGICAL POSITIONING OF THE PATIENT IN THE PERI-OPERATIVE AND PERI-ANAESTHESIOLOGICAL CONTEXT**
Competency 5<br>Admit the assisted person in the peri-operative and peri-anaesthesiological	**Learning outcome 1**<br>Admit the assisted person within the structural, environmental and organisational peri-operative and peri-anaesthesiological context, using the dedicated spaces and pathways, proceeding with the verification of the documentation and all the information recommended for the registration of the assisted person within the information system, taking care of the relationships established with the assisted persons
	**Activities/tasks**

context.	1. Review the operative notes to gather information about the assisted person, the surgical procedure, and the anaesthesiological conduct;<br>2. Receive the assisted person in the preparation room, operating theatre, and recovery room; greet them, introduce yourself, and explain the peri-operative and peri-anaesthesiological environment;<br>3. Ensure the documentation completeness for the surgical procedure, anaesthesiologic conduct, and the nursing care, including informed consents for instrumental and laboratory tests, gathering all necessary information also through observation and interviews.<br>4. Ensure that the assisted person's health conditions allow proceeding with the planned surgical procedure and the agreed-upon anaesthesiologic conduct;<br>5. Record the admission of the assisted person (date and time) and update the necessary information/data each time, using the documentation systems available;
	**Learning outcome 2**<br>Proceeding through observation, dialogue and interview to take charge of the assisted person's care needs, informing them about the nursing services that will be provided and formalising the care pact, verifying together with the OR team, in addition to identity, all the recommended safety checks, taking care of the relationships established with the human resources involved
	**Activities/tasks**
	1. Verify in the preparation room and in the operating theatre the patient's identity, the surgical site, the procedure, and the agreed-upon anaesthetic management. Check if the surgical site has been marked and if the preparation for surgery and anaesthesia in the operating unit has been completed. Gather information regarding fasting, administration of pharmacological therapy, removal of jewellery and removable prostheses. In the recovery room, verify the identity of the assisted person, the type of surgical procedure performed, the type of anaesthetic management adopted, the diagnostic procedures performed, the therapeutic procedures applied, and the positioning of medical devices;<br>2. Take charge of the care needs of the assisted person in the preparation room, operating theatre, and recovery room, considering the information and data acquired from the operative notes and specific documentation regarding anaesthetic management, surgical procedure, and related procedures performed and to be performed. Proceed with the planning of nursing care in all its phases: assessment, diagnosis, determination of outcomes, interventions, implementation, and evaluation of outcomes;<br>3. Inform the patient about all nursing services that will be performed during the perioperative and peri-anaesthetic period and formalise the care agreement, taking into account the expressed consent/disagreement, criteria of appropriateness, transparency, correctness, and compliance with current regulations on the protection of personal data;<br>4. Work, cooperate, and collaborate with the anaesthetist to verify that anaesthesia safety checks have been completed;<br>5. Operate, cooperate, and collaborate with the operating theatre team in identifying risks to the patient related to specific nursing activities involving instrumentation and anaesthesia (difficulty managing airways and/or aspiration, infectious risk and/or risk of allergic reactions, risk of deep vein thrombosis (DVT), risk of postoperative nausea and vomiting (PONV), risk of pain onset, risk of blood loss, risk of physical and skin injuries (including corneal, pressure, friction, and slipping injuries), risk of anxiety onset, risk of thermal disturbances (hypothermia and hyperthermia), radiation risk, and electrical risk);<br>6. Complete the nursing section of the operating theatre checklist (sign-in);
**Competency 6**<br>Transfer and position the assisted person in the peri-operative and peri-anaesthesiological context	**Learning outcome 3**<br>Transfer the assisted person into the peri-operative and peri-anaesthetic setting, using dedicated spaces and pathways, and ensuring the necessary materials and technological resources are available. Proceed with the operating theatre team to position the assisted person for surgery using recommended practices, fostering multiprofessional communication and cooperation.
	**Activities/tasks**
	1. Work, cooperate, and collaborate with colleagues and the entire team in selecting the operating table on which to transfer the patient, based on the surgical procedure to be performed;<br>2. Work, cooperate, and collaborate with the entire team in selecting the necessary medical devices (anti-decubitus mattresses, arm supports, shoulder supports, leg supports, belts, etc.) based on the anaesthetic management and surgical procedure, ensuring their integrity and functionality, and optimizing the operating table as required;

	3. Work, cooperate, and collaborate with colleagues from the wards or intensive care units, as well as the entire operating theatre team, during all patient transfers: from the bed to the transport system, from the transport system to the operating table, from the operating table back to the transport system, and from the transport system to the ward bed throughout the peri-operative and peri-anaesthetic period. This includes when the patient arrives from the ward or intensive care unit to the operating theatre and is transferred to the preparation room, the designated operating theatre, and the recovery room, and subsequently discharged back to the ward or intensive care unit, ensuring the functionality of all necessary medical devices as required;<br>4. Work, cooperate, and collaborate with the entire team in positioning the patient on the operating table, considering the various nursing, anaesthetic, and surgical procedures required throughout the perioperative and peri-anaesthetic period. Ensure the assisted person is positioned appropriately on the operating table to minimize interference with vital functions. Verify the proper functionality of the monitoring system and regularly check vital signs, common clinical signs, and symptoms through physical examination (as appropriate), and positions assumed throughout the peri-operative and peri-anaesthetic period. Prevent risks such as accidental falls, trauma, and physical and skin injuries (from pressure, friction, or slipping);<br>5. Promote the comfort of the assisted person and ensure privacy during all transfers and positioning, always considering the patient's mobility and joint freedom, as well as their level of understanding and cooperation;
**ACTIVITY AREA 3**	**PREPARATION OF THE ASSISTED PERSON FOR SURGICAL INTERVENTION AND ANAESTHETIC CONDUCT.**
**Competency 7**<br>Prepare the assisted person for the surgical procedure.	**Learning outcome 1**<br>Utilize dedicated spaces and pathways in the perioperative setting, along with necessary material and technological resources, verifying the pre-operative preparation carried out by colleagues. Proceed with the operating theatre team to conduct all safety checks, prepare the assisted person for the surgical procedure, and set up the surgical field according to recommended practices, cooperating with the surgical team.
	**Activities/tasks**
	1. Operate and cooperate with colleagues and the entire team to verify that the procedures listed in the Checklist related to preparing the assisted person for surgical intervention (and for anaesthetic management), especially where the person has not been able to fully or partially manage independently, have been performed in the Ward Unit. Upon entering the Operating Block, within the competence of the surgical team, ensure that the checked items correspond to the actual conditions required for access (as detailed in the full version of the document);<br>2. Verify in the preparation room the marking of the surgical site and laterality, where the type of surgical procedure requires it;<br>3. Operate and cooperate with the entire operating theatre team in applying the neutral electrode;<br>4. Work and cooperate with the entire operating theatre team to prepare the assisted person for safe and functional surgical positioning on the operating table, arranging the necessary medical devices (anti-decubitus, arm supports, shoulder supports, leg supports, belts, etc.);<br>5. Proceed with the surgical site antisepsis throughout the entire operative area of interest and ensure drying according to the antiseptic product used, cooperating with colleagues and collaborating with the surgical team;<br>6. Work and cooperate with the surgical team in positioning the transparent adhesive membrane (where used)<br>7. Work and cooperate with the surgical team in the proper setup of the surgical field, functionally applying surgical drapes and any other necessary medical devices on the assisted person during the procedure;<br>8. Work, cooperate, and collaborate in health education by providing information and guidance to the person (or to the family/caregivers of children or vulnerable individuals undergoing surgery) regarding behaviours to adopt in the ward during the peri-operative period;
**Competency 8**<br>Preparing the assisted person for anaesthesia conduct	**Learning outcome 2**<br>Utilise dedicated spaces and pathways in the peri-anaesthetic setting, as well as the necessary material and technological resources. Verify the pre-anaesthetic preparation carried out by colleagues, then proceed with the operating theatre team to check all safety controls. Prepare the patient for anaesthesia conduct and position all necessary devices for induction, maintenance, and recovery, following the recommended practices, in cooperation with the anaesthetic team.

	**Activities/tasks**
	1. Work and collaborate with colleagues and the entire team to ensure that the procedures listed in the checklist for preparing the patient for anaesthesia management (and for the surgical procedure), especially when the patient is unable to do so independently or partially independently, have been completed in the Unit. Upon entering the Operating Theatre, the anaesthetic team ensures that the checked items correspond to the actual conditions required for entry (as detailed in the complete version of the document);<br>2. Verify the laterality of the surgical site in the preparation room, where the type of surgical procedure requires it;<br>3. Work and collaborate with the entire operating theatre team in the application of the neutral electrode;<br>4. Work, cooperate, and collaborate with the anaesthetic team in preparing the patient for anaesthesia management, according to the type of surgical procedure, both in the preparation room and the operating theatre. Position all necessary medical devices appropriate to the planned procedures, in line with the agreed anaesthesia management and the identified surgical procedure (peripheral venous catheter, central venous catheter, invasive arterial pressure monitoring, consciousness monitoring, etc.);<br>5. In the preparation room, the designated operating theatre, and the recovery room, proceed with basic and/or specific peri-anaesthetic monitoring of the patient, depending on the agreed and executed anaesthesia management and the scheduled surgical procedure, as well as in relation to the necessary medical devices;<br>6. Work and cooperate with the anaesthetic team in preparing the patient for the necessary and appropriate postures to maintain during the procedures, based on the agreed type of anaesthesia;<br>7. Work and cooperate with the entire operating theatre team in preparing the patient for safe and functional surgical positioning on the operating table, arranging the necessary medical devices (anti-decubitus cushions, arm supports, shoulder supports, leg supports, belts, etc.);<br>8. Work, cooperate, and collaborate in providing information and guidance to the patient (or to the family members/caregivers of children or vulnerable individuals undergoing anaesthesia) about the behaviours to adopt in the operating theatre during the peri-anaesthetic period;
**ACTIVITY AREA 4**	**DELIVERY AND MANAGEMENT OF PERI-OPERATIVE AND PERI-ANAESTHESIOLOGICAL NURSING CARE IN BOTH ROUTINE AND EMERGENCY SETTINGS**
**Competency 9**<br>Managing perioperative nursing care	**Learning outcomeme 1**<br>Access and navigate the relevant operating theatre, positioning oneself next to the patient, the surgical team, and the surgical field. Proceed with the nursing instrumentation activities using the necessary material and technological resources, as well as the recommended behaviours, assessments, treatments, and interventions. Navigate the entire surgical procedure in a routine setting
	**Activities/tasks**
	1. Access the designated operating theatre, respecting the spaces and times provided during instrumentation activities;<br>2. Provide perioperative care education during the pre- and post-operative phases through discussions with the patient and/or significant others, informing the patient about all nursing interventions, services, and procedures that will be carried out. Explain the emotions that may be experienced following the surgical procedure and the conditions in which they may find themselves afterward, ensuring there are no unrealistic expectations. Additionally, promote a proactive health culture, prevention of complications, and post-operative rehabilitation;<br>3. Operate, cooperate, and collaborate in the application of the surgical safety checklist (according to guidelines), checking all items of the Sign In (as detailed in the full version of the document);<br>4. Carry out all assessments, both in the preparation room and in the operating theatre, before and during the surgical procedure, by operating, cooperating, and collaborating with colleagues and the surgical team, as also outlined in the surgical safety checklist (detailed in the full version of the document);<br>5. Operate and cooperate to ensure the presence and completeness of the general hospitalisation documentation, the specific surgical intervention documentation, and the nursing documentation, including informed consents (anaesthetic, surgical, blood components);<br>6. Operate, cooperate, and collaborate in the application of the surgical safety checklist (according to guidelines), checking all items of the Time Out (as detailed in the full version of the document);

	7. Ensure the neutral electrode of the electrosurgical unit is present and correctly positioned before the skin incision;<br>8. Gather information regarding additional medical devices and electromedical equipment necessary during the surgical procedure, based on Protocols, Procedures, and Operating Instructions relevant to the context and the surgical team's requirements;<br>9 Position yourself next to the patient, the surgical field, and the surgical team in a manner conducive to the surgical procedure, and connect the medical devices present in the surgical field to the electromedical equipment, in collaboration with colleagues present in the Operating Theatre;<br>10. Proceed directly in the surgical field with the planned nursing instrumentation activities, through treatments and interventions in line with the necessary evaluations and surgical procedures as they arise, and especially consistent with the risks that are effectively identified in the Operating Theatre. Navigate the course of the entire surgical procedure (from skin incision to closure), operating, cooperating, and collaborating with colleagues and the surgical team, respecting the different surgical phases (clean and contaminated), therapeutic procedures, and diagnostic procedures. Ensure the correct functioning of medical devices and electromedical equipment, using and counting instruments, gauze, cotton balls, needles, and sharps;<br>11. Monitor the access to the Operating Theatre and the movements of all team members to ensure the maintenance of asepsis throughout the entire surgical procedure (surgical field, instrument tables, etc.);<br>12. Operate, cooperate, and collaborate in the correct management of biological material. Operate, cooperate, and collaborate in the application of the surgical safety checklist by checking all items of the Sign Out (as detailed in the full version of the document);
	**Learning outcomeme 2**<br>Access and navigate the relevant operating theatre, positioning oneself next to the patient, the surgical team, and the surgical field. Proceed with nursing instrumentation activities using the necessary material and technological resources, as well as the recommended behaviours, assessments, treatments, and interventions. Navigate the entire surgical procedure in an emergency setting.
	**Activities/tasks**
	1. Proceed directly in the surgical field with nursing instrumentation activities in emergency situations, through treatments and interventions in line with the necessary surgical procedures as they arise, respecting the different surgical phases (clean and contaminated), evaluations, and identified risks. Navigate the course of the surgical procedure, manage and verify the correct functioning of medical devices and electromedical equipment, operate, cooperate, and collaborate in the application of therapeutic procedures and the execution of diagnostic procedures (as detailed in the full version of the document);
**Competency 10**<br>Manage perianaesthesiological nursing care	**Learning outcome 3**<br>Access and orient oneself in the preparation room, the designated operating theatre, and the recovery room, positioning oneself next to the patient and the anaesthesiology team. Proceed with anaesthesia nursing activities using the necessary material and technological resources, as well as the recommended behaviours, assessments, monitoring, treatments, and interventions. Navigate the course of the entire anaesthesiological procedure in a routine setting.
	**Activities/tasks**
	1. Access the designated Operating Theatre, respecting the spaces and times allocated during the anaesthesiological procedure;<br>2. Provide education on peri-anesthesiological care in the pre- and post-anaesthesiological phases, through discussions with the patient and/or significant others, informing them about all nursing interventions, services, and procedures that will be performed. Explain the emotions that may arise following the anaesthesiological procedure and the conditions they may experience afterward, ensuring there are no unrealistic expectations. Additionally, promote a proactive health culture, prevention of complications, and post-anaesthesiological rehabilitation;<br>3. Operate, cooperate, and collaborate in the application of the surgical safety checklist (according to guidelines), checking all items of the Sign In (as detailed in the full version of the document);<br>4. Carry out, both in the preparation room and in the operating theatre and recovery room, before, during, and after the anaesthetic procedure, performing, cooperating, and

	collaborating with colleagues and the anaesthetist, all evaluations, also referred to in the surgical safety checklist (complete in the full version of the document);<br>5. Work and cooperate by verifying that the general medical documentation of the hospitalization, the specific documentation of the surgical procedure and the anaesthetic conduct, and the nursing documentation, including informed consents (anaesthetic, surgical, blood components), are present and complete;<br>6. Operate, cooperate, and collaborate in the application of the surgical safety checklist, checking all the items of the Time Out (complete in the full version of the document);<br>7. Verify that the neutral electrode of the electrosurgical unit is present and correctly positioned before the skin incision;<br>8. Gather information regarding additional medical devices and electromedical equipment that are necessary during the anaesthetic procedure, based on Protocols, Procedures, and Operating Instructions outlined in the context of reference, and also based on the needs of the anaesthetic team;<br>9. Position yourself next to the patient and the anaesthetic team in a way that facilitates the anaesthetic procedure and apply the necessary medical devices and electromedical equipment, in collaboration with colleagues present in the preparation room, operating theatre, and recovery room;<br>10. Proceed with the planned nursing activities of anaesthesia through treatments and interventions that are in line with assessments, anaesthetic procedures that become necessary from time to time, and especially consistent with the risks that actually arise in the preparation room, operating theatre, and recovery room. Navigate the entire anaesthetic procedure (induction, maintenance, and recovery) by operating, cooperating, and collaborating with colleagues and the anaesthetic team in the application of therapeutic procedures (narcotic and non-narcotic medications, blood components, etc.) and in the execution of diagnostic procedures (blood tests, cytological exams, instrumental exams, etc.), managing and verifying the correct functioning of medical devices and electromedical equipment;<br>11. Operate, cooperate, and collaborate in the correct management of biological materials;<br>12. Operate, cooperate, and collaborate in the application of the surgical safety checklist by checking all the items of the Sign Out (complete in the full version of the document);
	**Learning outcome 4**<br>Access and orient yourself in the preparation room, the designated operating theatre, and the recovery room, positioning yourself next to the patient and the anaesthetic team. Proceed with the nursing activities of anaesthesia by utilising the necessary material and technological resources, and by applying the recommended behaviours, assessments, monitoring, treatments, and interventions, navigating the entire anaesthetic procedure under urgent and emergency conditions.
	**Activities/tasks**
	1. Proceed directly in the operating field with the essential nursing activities of anaesthesia in urgent and emergency situations, through treatments and interventions in line with the necessary anaesthetic procedures, the different surgical phases (clean and contaminated), the assessments, and the emerging risks. Navigate the course of the surgical intervention, managing and verifying the correct functioning of medical devices and electromedical equipment, operating, cooperating, and collaborating in the application of therapeutic procedures and the execution of diagnostic procedures (fully listed in the complete version of the document);
**ACTIVITY AREA 5**	**PROMOTION OF PERIOPERATIVE AND PERIANESTHETIC NURSING CARE CONTINUITY**
**Competency 11**<br>Promote perioperative care continuity	**Learning outcome 1**<br>Undertake initiatives aimed at promoting care continuity by verbally and in writing collecting all information regarding the planning of preoperative nursing care, proceeding with the planning of perioperative and postoperative nursing care, and verbally and in writing communicating the necessary interventions and the results achieved throughout the care pathway. This approach fosters the integration of nursing care with medical and surgical care, ensuring careful attention to the relationships established with the involved personnel.
	**Activities/tasks**
	1. Verify in the preparation room and/or in the operating theatre that the care continuity record is present in the general medical documentation of the hospitalization, specific to the surgical procedure and anaesthetic management, and in the nursing documentation;<br>2. Ensure that the care continuity record has been completed in all necessary parts, respectively in the ward (or in the Intensive Care Unit/Recovery Room or Emergency Department where applicable) and in the Preparation Room;

	3. Collect any missing data/information from colleagues in the ward (or in the Intensive Care Unit/Recovery Room or Emergency Department where applicable), employing effective communication methods and active listening techniques;<br>4. Verify that in the organizational settings (or in the Intensive Care Unit/Recovery Room or Emergency Department where possible) or in the Preparation Room, a general and/or specific nursing care plan for the perioperative pathway has been formulated, covering the pre-operative, intra-operative, and post-operative phases: assessment, formulation of diagnoses, determination of outcomes, and identification of interventions, implementation of interventions, and evaluation of outcomes;<br>5 Formulate the specific nursing care plan for the perioperative pathway, covering the pre-operative, intra-operative, and post-operative phases, respectively in the preparation room and/or operating theatre, taking into consideration the earlier planning and the level of satisfaction with the expected results;<br>6. Complete the care continuity record in all necessary parts, respectively in the Preparation Room and/or Operating Theatre;<br>7. Communicate to colleagues in the Operating Theatre or Recovery Room or Ward (and possibly in the Intensive Care Unit/Recovery Room), using effective communication methods and active listening techniques, all the necessary data and information related to the instrumentation nursing activities conducted (fully detailed in the full version of the document);
**Competency 12**<br>Promoting perianaesthetic care continuity	**Learning outcome 2**<br>Implement initiatives aimed at promoting continuity of care by collecting all information regarding the planning of pre-anaesthetic nursing care in both verbal and written form. Proceed with the planning of peri-anaesthetic and post-anaesthetic nursing care, transmitting the necessary interventions and the results achieved in both verbal and written form throughout the care pathway. Foster the integration between nursing care and medical-surgical care, taking care of the relationships established with the human resources involved.
	**Activities/tasks**
	1. Verify in the preparation room, operating theatre, and recovery room that the continuity of care form is present in the general hospital documentation, the specific documentation of the surgical procedure and anaesthetic management, and the nursing documentation;<br>2 Verify that the care continuity form has been completed in all necessary parts, respectively in the ward (or in the Intensive Care Unit or Emergency Department where possible), in the Preparation Room, and in the Operating Theatre;<br>3. Gather any missing data/information from colleagues in the ward (or the Intensive Care Unit or Emergency Department where possible), applying effective communication techniques and active listening skills;<br>4. Verify that in the organisational settings (or in Intensive Care or in the Emergency Department where possible), or in the Preparation Room or Operating Theatre, the planning of general and/or specific nursing care for the perioperative pathway has been formulated, covering all phases: assessment, diagnosis formulation, outcome determination and identification of interventions, implementation of interventions, and outcome evaluation;<br>5. Formulate the planning of specific nursing care for the peri-anaesthetic pathway, covering the pre, intra, and post-anaesthetic phases, respectively in the Preparation Room and/or Operating Theatre and/or Recovery Room, taking into account previous planning and the satisfaction level of the expected results;<br>6. Complete the continuity of care form in all necessary parts, respectively in the Preparation Room and/or Operating Theatre and/or Recovery Room;<br>7. Transmit to colleagues in the Operating Theatre, Recovery Room, or Ward (and potentially in Intensive Care), applying effective communication techniques and active listening skills, all necessary data and information related to the nursing anaesthesia activities carried out (fully reported in the complete document);
**ACTIVITY AREA 6**	**PROMOTION OF CLINICAL GOVERNANCE IN THE PERIOPERATIVE AND PERI-ANAESTHETIC SETTING**
**Competency 13**<br>Documenting planned and delivered nursing care in the perioperative setting	**Learning outcome 1**<br>Document in written form the planned perioperative nursing care in all its phases, completing the relevant sections of the safety checklist and other sections of the nursing documentation of interest. Clearly, comprehensively, accurately, and truthfully record the activities performed and services provided, the expected outcomes, and the level of outcomes achieved throughout the perioperative care pathway. Promote the integration between nursing care and medical-surgical care, and pay attention to the relationships established with the human resources involved.
	**Activities/tasks**

	1. Verify that the general hospital documentation, the specific documentation of the surgical procedure and anaesthetic management, and the nursing documentation are present and complete, including informed consents (surgical, anaesthetic, blood components) and instrumental and laboratory tests (indicate any missing documentation);<br>2. Complete, by working, cooperating, and collaborating with the entire team, the relevant parts of the operating theatre safety checklist (according to Guidelines) (fully reported in the complete document);<br>3. Use conceptual models, methods, tools, and a standardised and shared nursing language to document within the nursing record, both in the preparation room and in the operating theatre, the five phases of the process carried out (fully reported in the complete document). Specifically, write in a clear, complete, accurate, and truthful manner the part of the documentation for which you are responsible (fully reported in the complete document);<br>4. Document the planning of the nursing instrumentation activities carried out and subsequently, compared to what was planned, confirm or modify the perioperative nursing care provided – both in ordinary situations and in emergency situations (fully reported in the complete document);<br>5. Document the perioperative care education provided in the pre and post-surgical phases, including the informed consent or refusal to nursing treatment expressed by the patient;<br>6. Document the process of data and information transmission between the professional and the patient, and between one professional team and another, with the aim of preventing risks, ensuring the safety and appropriateness of care, and promoting its continuity;<br>7. Verify, manage, and archive the documentation used during the surgical procedure;
**Competency 14**<br>Documenting planned and delivered nursing care in the perianaesthetic setting	**Learning outcome 2**<br>Document in written form the planned peri-anaesthetic nursing care in all its phases, completing the relevant sections of the safety checklist and other sections of the nursing documentation of interest. Clearly, comprehensively, accurately, and truthfully record the activities performed and services provided, the expected outcomes, and the level of outcomes achieved throughout the peri-anaesthetic care pathway. Promote the integration between nursing care and medical-surgical care, and pay attention to the relationships established with the human resources involved.
	**Activities/tasks**
	1. Verify that the general hospital documentation, the specific documentation of the surgical procedure and anaesthetic management, and the nursing documentation are present and complete, including informed consents (surgical, anaesthetic, blood components) and instrumental and laboratory tests (indicate any missing documentation);<br>2. Complete, by working, cooperating, and collaborating with the entire team, the relevant parts of the operating theatre safety checklist (according to Guidelines) (fully reported in the complete document);<br>3. Use conceptual models, methods, tools, and a standardised and shared nursing language to document within the nursing record, both in the preparation room and in the operating theatre, the five phases of the process carried out (fully reported in the complete document). Specifically, write in a clear, complete, accurate, and truthful manner the part of the documentation for which you are responsible (fully reported in the complete document);<br>4. Document the planning of the anaesthesia nursing activities carried out and subsequently, compared to what was planned, confirm or modify the peri-anaesthetic nursing care provided – both in ordinary situations and in emergency situations – (fully reported in the complete document);<br>5. Document the peri-anaesthetic care education provided in the pre and post-surgical phases, including the informed consent or refusal of nursing treatment expressed knowingly by the patient;<br>6. Document the process of data and information transmission between the professional and the patient, and between one professional team and another, with the aim of preventing risks, ensuring safety and appropriateness of care, and promoting continuity;<br>7. Verify, manage, and archive the documentation used during the anaesthetic procedure;
**Competency 15**<br>Promoting perioperative Safety and Risk Management	**Learning outcome 3**<br>Adhere to recommended behaviours and best practices, adopting actions aimed at ensuring perioperative safety and risk management. Implement initiatives aimed at improving the quality of care delivered in the perioperative setting, following an integrated approach within the framework of Clinical Governance determinants.
	**Activities/tasks**

	1. Adopts behaviours aimed at ensuring peri-anaesthesia safety, according to an integrated approach between risk management and all other determinants of Clinical Governance; (a. Verify the safety of the peri-operative environment, i.e. of all furnishings, medical devices and electro-medical equipment used for surgery, including the verification of risks (present or potential), using checklists where present; b. Adhere to the recommendations referred to in the protocols, procedures, operating instructions and checklists, aimed at promoting the best safety conditions for the assisted person and operators through the prevention of risks, including infectious risks, and at reducing the occurrence of damage or adverse events related to healthcare services);<br>2. Operate, cooperate, and collaborate in the completion of the surgical safety checklist;<br>3. Monitor the microclimate in the preparation room, the reference operating theatre, and the recovery room. Adopt behaviours to maintain the microclimate and, if necessary, request adjustments from the relevant personnel. Where possible, make adjustments directly;<br>4. Carry out or request the perioperative cleaning and sanitisation of the environment, including all furnishings, electromechanical equipment, and reusable medical devices. Additionally, ensure that the processes have been carried out according to the guidelines and adopt behaviours that align with the functionality of these processes;<br>5. Operate, cooperate, and collaborate to ensure and maintain sterility during the execution of perioperative procedures, including the collection of documentation for the traceability and trackability of the sterile pathway;<br>6. Operate, cooperate, and collaborate to ensure and maintain latex-safe pathways during the execution of perioperative procedures;<br>7. Perform surgical handwashing before preparing the perioperative environment, continuously adhere to the prescribed times, and follow sterilisation principles throughout all procedures related to instrumentation, from setting up the operating table, main table, and side tables, to correctly dressing each member of the surgical team, from skin antisepsis to preparing the operative field (according to guidelines);<br>8. Ensure the disposal of non-hazardous medical waste, hazardous medical waste at risk of infection and non-infectious waste, as well as the disposal of waste requiring specific methods, in accordance with current legislative provisions (for example, disposal of paper, plastic, and glass according to urban waste management, disposal of gloves, gauze, syringes, needles, and sharps in appropriate non-reusable, rigid, hermetically sealed containers, plastic or otherwise, and disposal of batteries in designated containers);<br>9. Operate, cooperate, and collaborate with the surgical team in identifying patient risks associated with specific instrumentation activities (as detailed in the full version of the document) and adopt behaviours aimed at preventing and reducing these risks;<br>10. Operate, cooperate, and collaborate in preventing errors (ensuring safe movements of personnel present in the operating theatre during surgery, adherence to sterilisation principles for medical devices on the operative field, on side tables, and the main table during surgical activities and all procedures related to instrumentation, including initial, intermediate, and final cross-counting of surgical instruments, swabs, gauze, sponges, drapes, needles, and sharps used during surgery, recording them in the appropriate documentation according to protocols).<br>11. Operate, cooperate, and collaborate in the correct identification, recording, and preservation of collected biological material samples, including their subsequent dispatch to diagnostic services for necessary processes (according to guidelines, protocols, procedures, and operational instructions);<br>12. Operate, cooperate, and collaborate in the correct completion and preservation of nursing documentation (potentially integrated with specific documentation for the surgical intervention and anaesthetic management).
**Competency 16**<br>Promote Safety and Risk Management in the Perianaesthetic Environment	**Learning outcome 4**<br>Adhere to behavioural recommendations and best practices, adopting behaviours aimed at ensuring safety and risk management, implementing peri-anaesthetic initiatives aimed at improving the quality of care provided, according to an integrated approach aligned with the principles of Clinical Governance.
	**Activities/tasks**
	1. Adopt behaviours aimed at ensuring peri-anaesthetic safety, according to an integrated approach combining risk management with all other determinants of Clinical Governance:<br>a. Verify the safety of the peri-anaesthetic environment, including all furnishings, medical devices, and electromechanical equipment used for anaesthetic procedures, and assess existing or potential risks using checklists where available;<br>b. Adhere to the recommendations outlined in protocols, procedures, operational instructions, and checklists, aimed at promoting the best safety conditions for the patient and

	staff through the prevention of risks, including infections, and reducing the occurrence of harm or adverse events related to healthcare services;<br>2. Operate, cooperate, and collaborate in the completion of the surgical safety checklist;<br>3. Monitor the microclimate in the preparation room, the reference operating theatre, and the recovery room. Adopt behaviours to maintain the microclimate and, if necessary, request adjustments from the relevant personnel. Where possible, make adjustments directly;<br>4. Carry out or request the sanitisation and disinfection of the peri-anaesthetic environment, including all furnishings, electromechanical equipment, and reusable medical devices. Additionally, ensure that the processes have been carried out according to guidelines and adopt behaviours that align with the functionality of these processes.<br>5. Operate, cooperate, and collaborate to ensure and maintain sterility during the execution of peri-anaesthetic procedures, including the collection of documentation for traceability and sterility tracking;<br>6. Operate, cooperate, and collaborate to ensure and maintain latex-safe pathways during the execution of peri-anaesthetic procedures;<br>7. Perform antiseptic handwashing before preparing the peri-anaesthetic environment, continuously adhere to aseptic principles throughout all procedures related to anaesthetic management, from setup to arrangement of furnishings, medical devices, and electromechanical equipment, maintaining adherence to prescribed timelines (according to guidelines);<br>8. Ensure the disposal of non-hazardous medical waste, hazardous medical waste at risk of infection and non-infectious waste, as well as the disposal of waste requiring special handling, according to current legislative provisions (for example, disposal of paper, plastic, and glass following urban waste management guidelines, disposal of gloves, gauze, syringes, needles, and sharps in dedicated, non-reusable, rigid, sealed containers, plastic or otherwise, disposal of batteries in designated containers, and disposal of expired or unusable medications and/or psychotropic or narcotic substances, to be returned to the pharmacy using specific procedures);<br>9. Operate, cooperate, and collaborate with the anaesthetic team in identifying patient risks associated with specific anaesthesia activities (fully detailed in the complete document) and adopt behaviours aimed at preventing and reducing these risks;<br>10. Operate, cooperate, and collaborate in error prevention (procurement, storage, conservation, prescription, preparation, distribution, and administration of narcotic and non-narcotic medications, blood components, and blood derivatives, with particular emphasis on labelling of ready-to-use or reconstituted formulations following Good Preparation Practices or as comprehensive as possible in terms of component traceability such as active ingredient, dosage, preparation date, stability, photosensitivity, dilution for reconstituted formulations including reconstitution date, and all monitoring/control systems, etc.);<br>11. Operate, cooperate, and collaborate in the correct identification, recording, preservation, and dispatch to diagnostic services of collected biological material samples for necessary processes (according to guidelines, protocols, procedures, and operational instructions);<br>12. Operate, cooperate, and collaborate in the accurate completion and preservation of nursing documentation (potentially integrated into specific documentation for surgical interventions and anaesthetic management);
**Competency 17**<br>Promote professional development, skills enhancement, and best practices in perioperative and peri-anaesthetic settings	**Learning outcome 5**<br>Implement initiatives aimed at promoting Professional Development, Skills Enhancement, and Best Practices in perioperative and peri-anaesthetic contexts, adopting behaviours and approaches that integrate Clinical Governance determinants, Long Life Learning (Continuous, Permanent, Specialisation, and On-the-job Training), and the application of scientific research findings to clinical practice, contributing through active participation in research projects and improvement groups.
	**Activities/tasks**
	1. Plan and participate in training activities and fulfill obligations arising from the Continuous Medical Education programme, actively contributing to the construction of one's individual portfolio;<br>2. Seek training and act according to one's level of competence, seeking advice and intervention from experienced or specialist nurses for new activities or those with limited case experience whenever necessary;<br>3. Proactively commit to the continuous development of one's skills through study and the application of learning capabilities, critical thinking, and reflection based on experience;<br>4. Recognize the value of scientific research and experimentation and the safety of care as an integral part of the right to health;

	5. Contribute to the acquisition of knowledge validated by the Scientific Community to ensure the quality and safety of activities performed.<br>6. Adhere, with due consideration to specific circumstances, to the recommended behaviours outlined in guidelines developed by public and private entities, scientific societies, and technical-scientific associations of healthcare professions;<br>7. Adhere to best clinical practices in the absence of recommended behaviours outlined in guidelines, relying on evidence of effectiveness where available;<br>8. Actively and proactively participate in clinical, organizational, and educational research projects;<br>9. Actively and proactively participate in improvement groups for the development/review of operational tools (protocols, procedures, operational instructions, checklists, and Integrated Care Paths) and promote their continuous updating.<br>10. Makes research results available and actively engages in sharing, disseminating, and ensuring their correct application in various care settings;<br>11. Actively engages in basic professional education and training, providing guidance and support to Nursing Students from different degree programs who are entering the Operating Theatre environment;<br>12. Actively engages in basic professional education and training, providing guidance and support to Trainee Healthcare Assistants (OSS) from various educational institutions who are entering the Operating Theatre environment;<br>13. Actively engages in post-basic professional education and training, providing guidance and support to Nursing Students from different specialization courses, Masters programs, postgraduate degrees, and advanced training courses entering the Operating Theatre environment;<br>14. Actively and proactively engages in continuous and lifelong professional education and training, ensuring the learning accompaniment of all colleagues who are newly recruited, newly placed or in a training-on-the-job situation because they are involved in an advanced specialised skills development project through Job Rotation, Expanded Roles and Cross Fertilisation;

Notes: In the repertoire are not included knowledge, activities and skills. For th complete repertoire refer the the first author.
